# Dichotomic Role of Low-Concentration EGCG in the Oxaliplatin Sensitivity of Colorectal Cancer Cells

**DOI:** 10.1134/S160767292360029X

**Published:** 2024-01-08

**Authors:** Zhiyong Wang, Min Wang, Jiahao Huang, Mao Lin, Pei Wei

**Affiliations:** 1 Department of Immunology, Zhuhai Campus of Zunyi Medical University, Zhuhai, China; 2 Department of Pharmacy, Affiliated Hospital of Zunyi Medical University, Zunyi, China; 3 Department of Physiology, Zhuhai Campus of Zunyi Medical University, Zhuhai, China

**Keywords:** epigallocatechin-3-gallate, oxaliplatin, Vascular endothelial growth factor, colorectal cancer

## Abstract

Although epigallocatechin-3-gallate (EGCG) can potentiate chemotherapeutic drugs at high concentrations, its clinical translation is hampered by exceeding possible concentration thresholds. This study proposes a dichotomous use of low-concentration EGCG in chemotherapy. During the first cycle of combined treatment with oxaliplatin (OXA), low-concentration EGCG antagonized the cytotoxic effect of OXA on colorectal cancer (CRC) cells. However, when OXA was subsequently administered, the sensitivity of CRC cells markedly increased. Although low-concentration EGCG counteracted OXA, it reduced the OXA-induced secretion of vascular endothelial growth factor by tumor cells, thereby contributing to the increase in the sensitivity of tumor cells to the second round of OXA treatment. Therefore, low-concentration EGCG showed potential as a viable adjunct to modulate chemosensitivity in CRC.

## INTRODUCTION

As the third-most commonly diagnosed cancer and the second leading cause of cancer-related deaths worldwide, colorectal cancer (CRC) remains a major public health problem [1]. Despite remarkable advances in early detection methods and therapeutic strategies, the management of CRC remains an ongoing challenge, which is exacerbated by the emergence of chemotherapy resistance [2]. One of the main chemotherapeutic agents used to treat CRC is oxaliplatin (OXA), a platinum-based compound widely used for its cytotoxicity against tumor cells [3]. However, the emergence of drug resistance limits the potential of OXA for CRC therapy. Therefore, novel anticancer strategies should be explored and developed.

Numerous studies have focused on strategies to reverse chemotherapy resistance, particularly the combination of natural compounds [4]. An interesting natural candidate is epigallocatechin-3-gallate (EGCG), a prominent polyphenol discovered in green tea, which has been widely considered for its inherent anticancer properties [5]. EGCG may enhance the antitumor activity of chemotherapeutic drugs through various mechanisms [5]. However, its clinical applicability remains constrained because EGCG dosages used in most studies exceed the plasma concentrations observed following oral intake in humans [6]; as such, it has raised questions about the transferability of these findings to a clinical context. Additionally, high EGCG concentrations elicit in vivo toxicity, especially hepatotoxicity [7]. With these challenges, future studies should evaluate the therapeutic potential of lower EGCG concentrations in mitigating the OXA resistance of CRC cells.

## MATERIALS AND METHODS

### Reagents

The following reagents and materials were used in this study: fetal bovine serum (FBS, Inner Mongolia Opcel Biotech, Helingeer, China); MTT and LDH release kits (Keygenbio, Nanjing, China); EGCG (Tao Su Biotech Co., Ltd., Shanghai, China); OXA (Abmole, Shanghai, China); human VEGF ELISA kit (Abcam, Shanghai, China); rabbit anti-human VEGF antibody (4ABio, Beijing, China); mouse anti-human β-actin antibody (Santa Cruz Biotechnology, CA, USA); recombinant human VEGF165 (rhVEGF165) and human VEGF neutralizing antibody (R&D System, MN, USA).

### Cell Culture

Human CRC cell line HCT116 and HT29 were purchased from the Cell Bank of Chinese Academy of Sciences (Shanghai, China). Cells were incubated in DMEM supplemented with 10% FBS and 1% penicillin/streptomycin at 37°C in the presence of 5% CO_2_. Cellular strains were authenticated via short tandem repeat profiling. Mycoplasma was detected before each cell experiment.

### Cytotoxicity Assays

As described in our previous work [8], cell viability was evaluated using MTT assay in triplicate following the kit instruction. Absorbances were read immediately in a microplate reader using a test wavelength of 570 nm, with a reference wavelength of 620 nm.

### LDH Release Assays

The in vitro cell cytotoxicity was also evaluated by measuring lactate dehydrogenase (LDH) activity release from damaged cells into the supernatant. The experimental procedures were done in according with the manufacturer’s protocol as described in our previous work [8]. Absorbance was measured at a wavelength of 490 nm in a microplate reader. Total cellular LDH (high control) was determined by lysing the cells with 1% Triton X-100; the assay medium served as a low control and was subtracted from all absorbance measurements.

### In-Cell-Western

VEGF expression levels were determined by In-cell-western as previously described [9]. Briefly, CRC cells grown in 96-well plates (Jet Bio, Guangzhou) were incubated with 4.5 μM OXA for 6 h. Cancer cells were fixed, permeabilized, blocked, and incubated with rabbit anti-human VEGFA antibody (dilution 1 : 200) and mouse anti-human β-actin antibody (dilution 1 : 400). After washing and incubating with keyFluor 488 labeled anti-rabbit antibody (dilution 1 : 800) and keyFluor 594 labeled anti-mouse antibody (dilution 1 : 800), the microplate was scanned and analyzed with the ChemStudio PLUS Imaging System (Analytik Jena, Germany).

### ELISA Assays

ELISA kits were used to verify the VEGF levels in cell culture supernatants in accordance with the instructions provided by the manufacturer and a previous study [9]. Color intensity was assessed using a microplate reader (BioTek Instruments, Epoch) at 450 nm.

### Statistical Analysis

Experimental data were presented as the mean ± SE of three independent trials. Data were also statistically analyzed using *ANOVA* to investigate differences between groups. Results with *P* < 0.05 were deemed statistically significant.

## RESULTS

### Biphasic Modulation of OXA Sensitivity by Low-Concentration EGCG in CRC Cells

**Fig. 1.**
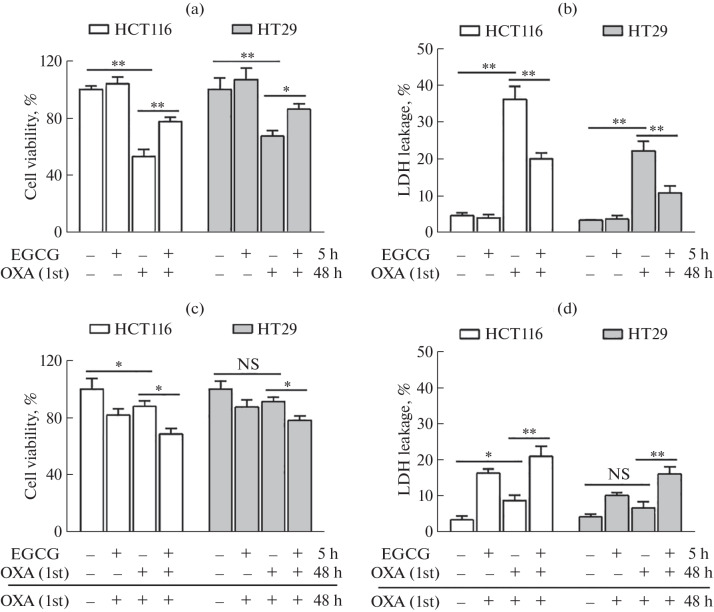
Dichotomous effect of low-concentration EGCG on the therapeutic sensitivity of OXA. (a and b) HCT116 and HT29 cells were pretreated with 6 μM EGCG for 5 h and then treated with OXA (4.5 μM) alone for 48 h. (a) Cell viability and (b) lactate dehydrogenase release were tested to evaluate the potential effects of EGCG on OXA-induced cytotoxicity. (c and d) HCT116 and HT29 cells were pre-treated with 6 μM EGCG for 5 h and then treated with OXA (OXA1st) for 6 h. After the first OXA treatment, cancer cells were washed twice, maintained in DMEM for 2 h, and re-treated with OXA (OXA2nd) for another 48 h. (c) Cell viability and (d) lactate dehydrogenase release were tested to evaluate the effects of EGCG on OXA2nd-induced cytotoxicity. Data are presented as means ± S.D. of three separate experiments; ^*^P < 0.05,^**^P < 0.01.

Oral EGCG administration of up to 800 mg/day is safe in adults [10). In addition, the range of maximum plasma concentration of EGCG after oral administration of a safe dose (50–800 mg/day) of EGCG is 0.16–6.11 μM, and the range of its half-life is 1.8–5.2 h [11]. Therefore, we chose 6 μM and 5 h as the working concentration and working time of EGCG for this experiment. We first focused on the effect of low-concentration EGCG on the sensitivity of colon cancer cells to 4.5 μM (correspond to the maximum plasma concentration) OXA. Surprisingly, cell viability assay results indicated that EGCG pretreatment did not enhance the cytotoxic effect of OXA (OXA1st) against colon cancer cells (Fig. 1a). Instead, it significantly countered the growth inhibitory effect of OXA. After the first treatment, we removed the drug and incubated the tumor cells in fresh medium for 2 h before further evaluating their responsiveness to the next round of OXA treatment, which helps to provide a more comprehensive understanding of the effects of OXA on tumor cells. Tumor cells were pre-treated with 6 μM EGCG for 5 h and then treated with OXA (OXA1st) for 6 h. After the first OXA treatment, tumor cells were washed twice, maintained in DMEM for 2 h, and re-treated with OXA (OXA2nd) for another 48 h. We found that, in the EGCG pretreatment group, the proliferation inhibition rates of HCT116 and HT29 cells markedly increased from 15 to 32% and from 9 to 21%, respectively, following an additional 48-h exposure to OXA (OXA2nd; Fig. 1c).

This intriguing observation suggested that pre-treatment with low-concentration EGCG might increase the sensitivity of colon cancer cells to subsequent OXA administration. Supporting these findings, our lactate dehydrogenase (LDH) release experiments were consistent with the results of the cell viability assays (Figs. 1b and 1d). These results substantiated the complex biphasic modulation of OXA sensitivity by low-concentration EGCG in CRC cells.

### Enhanced Sensitivity to OXA Retreatment Due to the Altered Secretory Profile of CRC Cells Induced by Low-Concentration EGCG

We hypothesized that the observed increase in sensitivity to a subsequent round of OXA treatment (OXA2nd) might be due to changes induced in the culture medium after low-concentration EGCG treatment. To test this hypothesis, we treated tumor cells with EGCG and OXA sequentially as described previously.

**Fig. 2.**
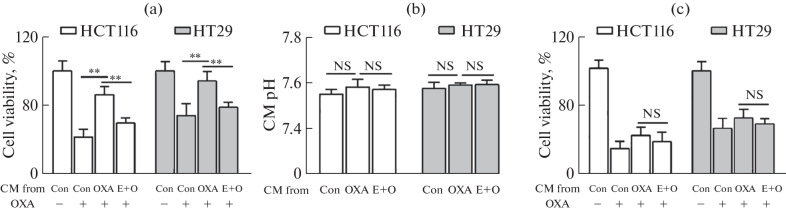
Low-concentration EGCG increased OXA2nd sensitivity by altering the secretory profile of CRC cells. (a) Effects of different conditioned medium (CM) on cancer cell viability after exposure to OXA for 48 h. (b) pH of different CM was measured using a pH meter. (c) Effects of different repeated freeze-thawed (F-T) CM on cancer cell viability after exposure to OXA for 48 h. Data are presented as means ± S.D. of three separate experiments; ^*^P < 0.05, ^**^P < 0.01.

After OXA treatment for 6 h (did not affect cell viability, data not shown), we removed the drugs and incubated the cells in a fresh medium for another 48 h to obtain the conditioned media (CM). After exposing the untreated tumor cells to OXA-containing CM, we found that the sensitivity of tumor cells to OXA was significantly enhanced in the CM derived from the co-treatment with low-concentration EGCG and OXA compared with that in the CM obtained from OXA treatment alone (Fig. 2a). Considering that changes in CM may reflect variations in composition and pH, we measured the pH of the different CM and found no significant differences between the groups (Fig. 2b). Therefore, the sensitizing effect of the CM on OXA might be attributed to changes in the CM composition. Proteins secreted by tumor cells into a medium can influence their own properties and those of other tumor cells. Therefore, we further postulated that changes in protein molecules within the CM could alter the sensitivity of tumor cells to re-treatment with OXA. To investigate this hypothesis, we subjected the different CMs to three freeze-thaw cycles to denature the protein contents. Remarkably, this procedure significantly abolished the disparity in sensitivity to a second round of OXA treatment between tumor cells grown in different CM (Fig. 2c). This finding supported our hypothesis that shifts in the protein profile within the CM might cause the differential responsiveness of tumor cells to OXA re-treatment.

### Effect of the Inhibition of VEGF Secretion by Low-Concentration EGCG on the Sensitivity of CRC Cells to OXA Re-treatment

Given the current clinical use of aflibercept, a drug that targets VEGF, for the treatment of CRC resistant to OXA-based regimens [12], we hypothesised that changes in VEGF levels in the culture medium might be an important contributor to the difference in sensitivity between initial and subsequent treatment with OXA.

**Fig. 3.**
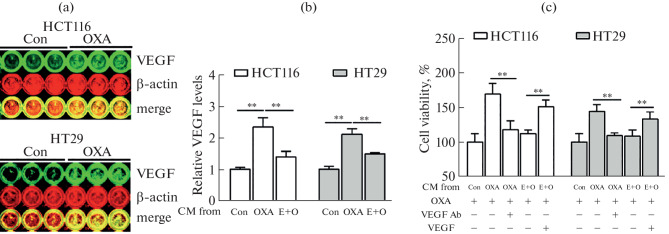
Low-concentration EGCG increased OXA2nd sensitivity by inhibiting the VEGF secretion of CRC cells. (a) HCT116 and HT29 cells were treated with 4.5 μM OXA for 6 h, the VEGF expression levels were detected by In-cell-western. (b) VEGF levels of different CM was measured via ELISA. (c) The effects of different CM containing a VEGF neutralizing antibody (1 μg/mL) or rhVEGF165 (2.5 ng/mL) on CRC cell viability were investigated after exposure to 4.5 μM OXA for 72 h. Data are presented as means ± S.D. of three separate experiments; ^*^P < 0.05, ^**^P < 0.01.

We thus examined the levels of VEGF after the 1st OXA treatment by in-cell-western and found that OXA significantly induced VEGF expression in CRC cells (Fig. 3a). We therefore hypothesized that oxaliplatin could similarly promote VEGF secretion. ELISA results showed that OXA indeed induced an increase in VEGF secretion in CRC cells (Fig. 3b). Furthermore, ELISA revealed that low-concentration EGCG could effectively suppress OXA-induced VEGF secretion in CRC cells (Fig. 3b). Furthermore, when untreated tumor cells were incubated in OXA-containing CM, the addition of exogenous VEGF (2.5 ng/mL) to the CM obtained after co-treatment with low-concentration EGCG and OXA caused a decrease in tumor cell sensitivity to 2nd OXA treatment (Fig. 3c). Meanwhile, the addition of VEGF-neutralizing antibody (1 μg/mL) to OXA-treated CM restored tumor cell sensitivity to OXA re-treatment (Fig. 3c).

## DISCUSSION

The potential of EGCG as a potent chemotherapeutic enhancer has been recognized in the field of oncology research [13]. However, its clinical application is limited because it exceeds possible concentration thresholds (0.16–6.11 μM) [11]. Therefore, alternative approaches that involve using lower EGCG concentrations and still improve chemotherapy outcomes should be developed. In this study, we demonstrated the biphasic role of low-concentration EGCG in modulating the sensitivity of CRC cells to OXA.

Previous research showed that EGCG is a promising chemosensitizing agent, but the clinical translation of these findings remains elusive because of unachievable doses and potential toxicity issues. For example, to achieve a plasma concentration of 50 µM EGCG, an adult should consume approximately 500 cups (240 mL/cup) of green tea per day [14], which is an unrealistic expectation. In our study, while an in vitro dosage of 6μM EGCG may reflect a high plasma concentration, it is also possible to achieve this concentration in the gut. In addition, safety concerns associated with high EGCG doses have been raised. Clinical studies have shown that high doses of EGCG can cause liver toxicity, nausea, abdominal pain, diarrhoea and other clinical symptoms [15, 16]. In addition, animal studies have shown that the effective dose of EGCG is very close to the toxic dose [17]. Therefore, lower and safer doses must be established for the clinical use of EGCG. Our study changed the narrative by revealing an underexplored facet of EGCG, which is its dichotomous role at low concentrations. We found an initial antagonistic effect of low-concentration EGCG on OXA cytotoxicity and an unexpected potentiating effect in subsequent treatments. Importantly, this novel finding aptly reflected the clinical process of OXA resistance in patients. That is, drug resistance in tumor cells does not occur overnight; instead, it develops over time and with repeated exposure to the drug. Given this unique perspective, we should reconsider the viable strategy of using EGCG as an adjunctive treatment for CRC.

Our research highlighted the critical role of reduced VEGF secretion in increasing the sensitivity of CRC cells to the second round of OXA treatment following EGCG administration. VEGF is a potent mediator of angiogenesis, which promotes new blood vessel formation and plays an indispensable role in tumor progression and metastasis [18]. Importantly, increased VEGF levels are associated with chemotherapy resistance in several cancers, including CRC. For example, an increased VEGF expression can protect tumor cells from apoptosis induced by chemotherapeutic drugs, thereby triggering chemoresistance development [19]. This protective role of VEGF extends not only to promoting angiogenesis, but also to directly upregulating anti-apoptotic pathways and proteins in tumor cells. For instance, VEGF inhibition can enhance the apoptosis sensitivity of HCT116 and LS174T CRC cells to 5-Fluorouracil in vitro by up-regulating BCL2 associated X protein (BAX) and down-regulating survivin [20]. Additionally, VEGF inhibitors can heighten the apoptosis sensitivity of hepatocellular carcinoma cells to etoposide in vitro through the suppression of protein kinase B (PKB/AKT) and extracellular signal-regulated kinase (ERK) pathways activation [21]. Although neutralizing antibodies or inhibitors of VEGF are commercially available, these drugs pose the risk of oversuppressing VEGF, which may limit the execution of its normal physiological functions and subsequently cause a series of side effects such as bleeding and gastric perforation [22]. In this regard, low-concentration EGCG may moderately suppress VEGF. These observations not only highlighted the dual role of EGCG in modulating chemosensitivity but also revealed a novel interface between dietary polyphenols and VEGF regulation in the context of chemotherapy.

Although our study provided insightful observations regarding the role of EGCG in modulating the sensitivity of CRC cells to OXA therapy, some limitations should be comprehensively considered. First, the specificity of our focus on CRC cells and the chemotherapeutic agents, OXA and EGCG, might limit the broad applicability of our findings to various cancer types and treatment protocols. The potential sensitizing role of low-concentration EGCG in various cancer types is promising and should be further investigated. Second, although our research lays a promising foundation, we should recognize the inherent complexity of malignant tumors, including the multiplicity of tumor components and the highly variable tumor microenvironment, which may affect the interactions of EGCG with tumor cells. Therefore, extensive investigations in a more complex physiological context should be performed to translate these findings into practical clinical applications. Third, we proposed that low-concentrations EGCG might cause fewer side effects than other vascular endothelial growth factor inhibitors; however, the potential side effects associated with this treatment strategy, including the long-term effects and sustainability of the combination of OXA and low-concentration EGCG, should be thoroughly investigated. Lastly, our study focused primarily on the modulation of VEGF levels by EGCG and might overlook other mechanisms by which EGCG likely affected tumor growth and chemosensitivity. Future research should investigate other molecular pathways affected by EGCG to extend our findings.

## CONCLUSIONS

In conclusion, our research provided promising insights into the potential of EGCG for enhancing the efficacy of OXA in CRC treatment. Future studies should further explore the wide range of molecular pathways that EGCG could affect to develop and expand its use. Thus, EGCG could be used as an effective compound in advancing cancer therapy.
